# Advancements and Challenges in Computational Biology

**DOI:** 10.1371/journal.pcbi.1004053

**Published:** 2015-01-08

**Authors:** Ruth Nussinov

**Affiliations:** 1 Cancer and Inflammation Program, Leidos Biomedical Research, Inc., Frederick National Laboratory for Cancer Research, National Cancer Institute, Frederick, Maryland, United States of America; 2 Sackler Institute of Molecular Medicine, Department of Human Genetics and Molecular Medicine, Sackler School of Medicine, Tel Aviv University, Tel Aviv, Israel

Computational biology has soared from being an auxiliary discipline to being a crucial element for progress in practically all aspects of the biological sciences. In this annual Editorial, I would like to step back, consider significant computational biology advances of the last decade, and reflect on some key challenges ahead. The timing is particularly appropriate. *PLOS Computational Biology*, the premier journal in computational biology, is approaching its tenth anniversary. The task is daunting; not only has the field come a long way in ten years but it is broad with many advances to consider. In addition, since computational biology has become closely tied to experimental research, progress is not purely computational; it is tied to experiment. And that's as it should be. Ten years ago, computational biology was not entirely trusted by experimental biologists. By contrast, today computational biology is integrated in the community. It's easier for computational biologists to collaborate across disciplines. Laboratory scientists have a better understanding of the merit of computational models for hypothesis generation as well as the need to iterate between modeling and laboratory testing [1].

We have witnessed huge leaps in biological computing [2]. We now have at our disposal large information-rich resources, and we are increasingly able to integrate and understand the vast quantities of data that they encompass. We have also made big strides toward multiscale biological modeling, and we have a vastly more networked world of researchers and their data. Analysis of massive gene expression and proteomic data permitted the construction of comprehensive and predictive models for cellular pathways, as well as software for inferring interaction networks, and steps toward modeling of cells. Genes susceptible to disease have been identified and, on a different level, the electrical behavior of neurons has been modeled. Molecules have been imaged in action and networks that regulate cell functions untangled. Matching targets for selective cancer therapy is difficult. Nonetheless, recent strategies have been proposed to restrict the combinatorial space, minimize toxicity, and increase the precision and power of such restrictive combinations, altogether leading to drugs that could be tested in clinical trials. Leveraging the enhanced identification of drug targets, including repertoires of redundant pathway combinations, has been helped by such innovative concepts [3].

Formidable challenges include: the establishment of computer networks for surveillance of disease; mapping the pathways and biological networks associated with the initiation, growth and spread of cancer; predicting function and mutational dysfunction in disease from the structure of complex molecules; resolving the mechanisms of oncogenic mutations and the cellular network which is rewired in cancer; achieving accurate, efficient, and comprehensive dynamic models; and moving from artificial intelligence to the “connectome”—the connections among all of the neurons of the brain. Multiscale biological modeling—an area where vast progress has been made during the last decade—still faces major challenges. To tackle this aim, hybrid methods across disciplines, scales, and sources are essential. Hybrid methods integrate data from, for example, serial crystallography and time-resolved wide-angle X-ray scattering, micro- and nano-crystals for (future) free-electron lasers, electron microscopy, fluorescence resonance energy transfer (FRET), cross-linking data, small-angle X-ray scattering, crystallography, nuclear magnetic resonance (NMR), and more. Equally important is the development of protocols for model validation. We may expect an influx of models based on experimental data integration. If these are to be deposited in a public archival system, which is now a community aim, such clear protocols are essential for maintaining quality control. Finally, studying the dynamics of large integrated models is increasingly used to improve our understanding of how large complexes function in the cell and how they are regulated. The dynamics of such large associations provides an additional hugely complex layer; to date, we are still struggling to comprehend the dynamics of single molecules and their associations. This is compounded by the fact that large regions of the molecules can be disordered, and multiple temporal post-translational modifications take place, with different combinations spelling distinct functions. On a different level, improved tumor mutational analysis platforms and knowledge of the redundant pathways, which can take over in cancer, may not only supplement known actionable findings but forecast possible cancer progression and resistance. Such forward-looking can be powerful, endowing the oncologist with mechanistic insight and cancer prognosis, and consequently more informed treatment options.

Lastly, the community faces the global challenge of linking genetics to phenotype, including the genetics of cancer. Genetics is mediated by dynamic conformational ensembles. Powerful ideas such as that of the free energy landscape [4], imported from physics and chemistry, can help solve the mysteries of life. Biomolecules are not static sculptures; they are dynamic objects that are always interconverting between structures with varying energies. Such ideas help to understand how and why one-dimensionally connected biomolecules can organize themselves into functionally relevant ensembles of three-dimensional conformation [5]. Designing high affinity drugs that work is yet another highly significant aim.

The significance of any research advance and challenge—achieved or aspired to—is a matter of opinion. The list above is partial, incomplete, and possibly biased toward structural biology and cancer. Nonetheless, this list does indicate the magnitude of the tasks confronting computational biology as a discipline. In the absence of a meaningful way to quantify a journal's contribution to a field, it is unclear whether, and to what extent, *PLOS Computational Biology* has contributed to each advance and challenge. Manuscripts can be declined, for example, because of the absence of substantiating experimental data at the time, lack of sufficient rigor, or if the manuscripts included new experimental data, the authors may have opted for alternative journals. At the same time, it may also suggest that *PLOS Computational Biology* needs to be more open and receptive to new concepts. Differentiating between novel ideas that may lead to key advances and speculative propositions can, however, be challenging.

*PLOS Computational Biology* aims to serve the biological community and welcomes manuscripts addressing all areas of computational biology. We encourage submission of research papers describing novel results that provide significant new insights into biological processes and of methods papers presenting new protocols for tackling key problems that have been shown, or have the promise to provide, new biological insights. We aspire to be the journal that will publish key computational advances in the next decade with the rigor that *PLOS Computational Biology* is known for. The *PLOS Computational Biology* editorial team seeks to identify and publish only the most outstanding papers, aiming to consider only those that are of exceptional quality. Our goal of furthering our understanding of living systems through the application of computational methods is shared with the International Society for Computational Biology (ISCB); together, we hope to meet the challenge.

Finally, for 2015, our tenth anniversary year, *PLOS Computational Biology* plans to publish a series of “Focus Features” addressing key areas of computational biology. We welcome suggestions from our community.

## References

[1] Nussinov R (2014) A top 12 list for Biocomputing. A decade of progress and challenges ahead. Biomedical Computation Review 17–22.

[2] Jakobsson E (2005) The top ten advances of the last decade. Biomedical Computation Review 11–15.

[3] Nussinov R, Jang H, Tsai CJ (2014) The structural basis for cancer treatment decisions. Oncotarget 5: 7285–7302. 25277176 10.18632/oncotarget.2439 PMC4202123

[4] Frauenfelder H, Sligar SG, Wolynes PG (1991) The energy landscapes and motions of proteins. Science 254: 1598–1603. 1749933 10.1126/science.1749933

[5] Nussinov R, Wolynes PG (2014) A second molecular biology revolution? The energy landscapes of biomolecular function. Phys Chem Chem Phys 16: 6321–6322. 24608340 10.1039/c4cp90027h

